# Burden of disease of respiratory syncytial virus in infants, young children and pregnant women and people

**DOI:** 10.14745/ccdr.v50i12a01

**Published:** 2024-01-01

**Authors:** Elissa M Abrams, Pamela Doyon-Plourde, Phaedra Davis, Nicholas Brousseau, Andrea Irwin, Winnie Siu, April Killikelly

**Affiliations:** 1 Centre for Immunization Programs, Public Health Agency of Canada, Ottawa, ON; 2 University of Manitoba, Department of Pediatrics, Section of Allergy and Clinical Immunology, Winnipeg, MB; 3 University of British Columbia, Department of Pediatrics, Division of Allergy and Immunology, Vancouver, BC; 4 University of Ottawa, School of Epidemiology and Public Health, Ottawa, ON; 5 Institut national de santé publique du Québec, Québec, QC; 6 Yukon Communicable Disease Control, Health and Social Services, Government of Yukon, Whitehorse, YT

**Keywords:** respiratory syncytial virus, infants, burden of disease, surveillance, epidemiology

## Abstract

**Background:**

Passive immunization products for infants and pregnant women and people have sparked interest in understanding Canada’s respiratory syncytial virus (RSV) burden. This rapid review examines RSV burden of disease in infants, young children and pregnant women and people.

**Methods:**

Electronic databases were searched to identify studies and systematic reviews reporting data on outpatient visits, hospitalizations, intensive care unit admissions, deaths and preterm labour associated with RSV. We also contacted Canadian respiratory virus surveillance experts for additional data.

**Results:**

Overall, 17 studies on infants and young children and 10 studies on pregnant women and people were included, in addition to primary surveillance data from one Canadian territory (Yukon). There were higher rates of medical utilization for infants than older children. Hospitalization rates were highest in infants under six months (more than 1% annually), with 5% needing intensive care unit admission, but mortality was low. Severe outcomes often occurred in healthy full-term infants and burden was higher than influenza. Respiratory syncytial virus attack rate was 10%–13% among pregnant women and people. Only one study found a higher hospitalization rate in pregnant women and people compared to non-pregnant women and people. Limited evidence was found on intensive care unit admission, death and preterm birth for pregnant women and people.

**Conclusion:**

While risk of severe outcomes is larger in high-risk infants and children, healthcare burden is greatest in healthy term infants. The RSV severity for pregnant women and people appears to be similar to that for non-pregnant women and people.

## Introduction

Respiratory syncytial virus (RSV) is a common respiratory virus, affecting nearly all children younger than two years of age ((1)). Globally, RSV contributes to 31% of pneumonia cases, causing 33 million acute respiratory infections (ARI), 3.1 million hospitalizations and 118,200 deaths annually ((2)). Respiratory syncytial virus ranks as the third leading cause of lower respiratory deaths in children younger than five years of age, after *Streptococcus pneumoniae* and *Haemophilus influenzae* type b ((3)).

The RSV vaccine landscape has evolved. Previously, only one passive immunization product (palivizumab; a monoclonal antibody) was available for high-risk infants. Canada anticipates at least two new products; nirsevimab, a long-acting monoclonal antibody, and a RSV stabilized pre-fusion subunit protein vaccine for pregnant women and people (Pfizer RSVpreF vaccine, Abrysvo), offering both active and passive immunity for newborns. As the indication for the new passive immunization product includes healthy infants, and as the vaccine for pregnant individuals would protect both healthy and higher-risk infants, there is a need for an understanding of RSV’s burden in infants, young children and pregnant women and people.

Throughout this article we will refer to “pregnant women and people” and intend it to be an inclusive term to include people of all gender identities who are pregnant. We recognize this language is evolving and our aim is to use language that removes barriers to care.

While a recent review focused on high-risk infants (including prematurity, cardiopulmonary disease and immunocompromised), less data exists on RSV’s burden in healthy infants and young children in Canada ((4)). To inform recommendations for RSV prevention, we conducted literature reviews on RSV’s burden focusing on healthy infants (younger than 12 months of age) and young children (12–24 months of age). Since one approach involves vaccinating pregnant women and people, we also explored RSV’s burden in this group. This rapid review aims to summarize the available evidence on RSV burden of disease in infants, young children and pregnant women and people in Canada and other high-income countries.

## Methods

### Search strategies

Three search strategies were developed by a research librarian from Health Canada and the Public Health Agency of Canada. One focused on systematic reviews (SR) of RSV burden in infants and young children (**Supplemental material S1**). Two targeted RSV burden in pregnant women and people, with one concentrating on primary evidence studies and the other on systematic reviews (**Supplemental material S2**). Embase, MEDLINE, Global Heath and ProQuest Public Health databases were searched for studies published from January 1, 1995, to April 10, 2023. We also contacted Canadian respiratory virus surveillance experts for additional data. After removal of duplicates, references were uploaded in DistillerSR online software (Evidence Partners, Ottawa, Ontario).

### Study selection

Two reviewers (for pregnant women and people and for infants and young children) screened titles and abstracts for study eligibility. The articles pertaining to infants and young children focused on healthy infants younger than 12 months and healthy young children 12–24 months of age but did not exclude articles that captured high-risk infants. Full texts of selected articles were then evaluated. A second independent reviewer assessed citations marked for exclusion, with disagreements resolved through discussion. The reference lists of included studies were also screened for relevant articles on RSV burden in high-income countries including Canada and the United States (US) for infants and young children; due to a paucity of data, we did not restrict articles pertaining to pregnant women and people to high income countries.

### Eligibility criteria

Observational studies, randomized controlled trials (RCTs) and systematic reviews that met the criteria outlined in **Table 1** were included. Inclusion was limited to studies conducted after 1995 to capture the most recent evidence. The evaluation of RSV burden focused on clinical outcomes of interest in infants, young children and pregnant women and people and considered emergency department (ED) or outpatient visits, hospitalizations, intensive care unit (ICU) admissions, death and preterm labour associated with RSV.

**Table 1:** Study inclusion and exclusion criteria

PICOS	Inclusion	Exclusion
Population	Infants and children (focus on 24 months of age and younger) Pregnant women and people	Adults only
Intervention	N/A	N/A
Control	N/A	N/A
Outcome	Emergency department or other ambulatory visit due to RSV Hospitalization due to RSV-associated disease ICU admission due to RSV-associated disease RSV-associated death Preterm labour associated with RSV	Outcome not associated with RSV infection
Study design	Systematic reviews and/or meta-analyses Any primary evidence studies (i.e., experimental, quasi and non-experimental studies)	Narrative reviews Guidelines Editorials, commentaries Conference abstracts

Abbreviations: ICU, intensive care unit; N/A, not applicable; PICOS, population, intervention, control, outcome and study design; RSV, respiratory syncytial virus

### Data extraction and data synthesis

One reviewer extracted data from each article, verified by a second reviewer. Disagreements were resolved through discussion. Data included event number, sample size and effect measures. Results were synthesized narratively based on the study population and outcomes. Due to the value of Canadian data on RSV’s burden in healthy infants and young children, surveillance data from one territory (Yukon Communicable Disease Control) were included in this literature review.

## Results

### Infants and young children

**Figure 1:**
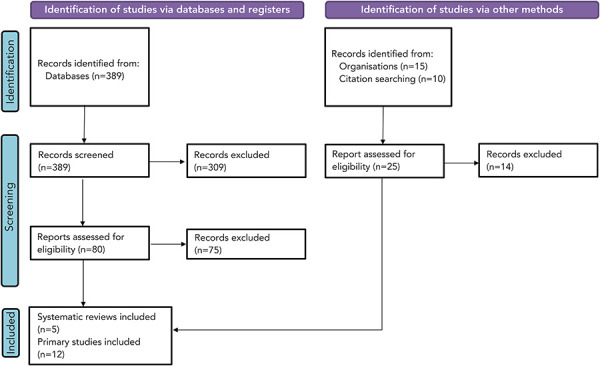
Study selection PRISMA flow diagram of infants and young children

**Study selection:** After deduplication, 389 references underwent screening (**Figure 1**). Seventeen articles, including five systematic reviews, were incorporated into the narrative synthesis of RSV burden in infants and young children (**Table 2**).

**Table 2:** Summary of included studies on the burden of disease of respiratory syncytial virus in infants and young children

Author, year, (reference), country	Study design	Study period	Population	Outcome definition	Results
**Medically attended RSV respiratory tract infection**					
Hall *et al.,* 2009 ((5)) US	Prospective population-based surveillance study (NVSN)	October 2000 to September 2004, during the winter months (November–April)	Children under five years of age and had received a diagnosis of acute respiratory infection (n=5,067)	Specimens were defined as positive if RSV was detected by viral isolation or by duplicate RT-PCR assays	Of 5,067 participants, 1,014 (20%) were treated in ED and 1,161 (23%) were treated in paediatric offices: • 919 (18%) were infected with RSV • 564 (61%) were hospitalized • 355 (39%) were outpatient • 184 (52%) were treated in ED • 171 (48%) were treated in paediatric office 18% of ED visits (184/1,014) and 15% of paediatric office visits (171/1,161) were RSV-associated
Bourgeois *et al.,* 2009 ((6)) US	Prospective cohort study	2003–2005	Children seven years of age and younger and treated in the ED for an acute respiratory infection (n=895)	Nasopharyngeal specimens were considered RSV-positive if RSV was detected through direct immunofluorescent antibody stain and/or RT-PCR	ED visit rates: • 10.2 per 1,000 ED visits attributable to influenza • 21.5 per 1,000 ED visits attributable to RSV Children 0–23 months: 64.4 ED visits per 1,000 attributable to RSV
Wildenbeest *et al.,* 2023 ((7)) Europe (Spain, Finland, England, Scotland and the Netherlands)	Multicentre, prospective birth cohort study	2017/07/01–2020/07/31 October 1 to May 31, parents were contacted weekly to reported ARI symptoms of their child	Healthy term infants, defined as children born at 37 weeks or more of gestation with no evidence of significant disorders^a^, were included in the active surveillance cohort (n=993)	A RSV-positive ARI episode was defined as a positive test result from either in-house RT-qPCR or POCT or both	Medically attended RSV-positive ARI: • Incidence: 14.1% (12.3–16.0), n=129 infants • Incidence rate per 1,000 infant-months: 12.1 (10.2–14.3), n=131 events RSV-positive ARI: • Incidence: 26.2% (24.0–28.6), n=249 infants • Incidence rate per 1,000 infant-months: 23.7 (21.0–26.7), n=262 events
Li *et al.,* 2022 ((8)) World Bank income regions (data reported for high income)	Systematic review of studies published 2017/01/01–2020/12/31	2019 or before (i.e., before the onset of the COVID-19 pandemic)	Children 0–60 months of age	RSV-associated acute lower respiratory infection was defined as acute lower respiratory infection with lab-confirmed RSV infection RSV-attributable acute lower respiratory infection was defined as acute lower respiratory infection that could be causally attributable to lab-confirmed RSV-infection	Incidence rate (UR) of RSV-associated acute lower respiratory infection in high-income regions (number of studies): • 0–3 months: 19.6 (6.5–59.7), n=3 • 3–6 months: 17.9 (4.8–66.7), n=3 • 0–6 months: 29.0 (12.9–65.0), n=4 • 6–12 months: 32.5 (19.9–53.0), n=4 • 0–12 months: 38.5 (21.6–68.8), n=5 • 0–60 months: 24.3 (13.8–42.7), n=7
**RSV respiratory tract infection with hospitalization**					
Schanzer *et al.,* 2006 ((9)) Canada	Retrospective population-based study	September 1994 to August 2000 (six influenza seasons, 1994/1995–1999/2000)	Hospitalized children younger than 19 years old	Diagnostic codes (ICD-9) selected based on their association with viral respiratory illness in children. RSV-attributable bronchiolitis admissions provided a better proxy for RSV activity than RSV positive specimens alone	RSV rates were highest in infants younger than six months of age at approximately 2,000 per 100,000
Papenburg *et al.,* 2012 ((10)) Canada (QC)	Prospective cohort study	Four consecutive winter seasons (2006/07–2009/10)	Children aged 0–35 months presenting as outpatients to paediatric clinic or hospitalized for RTI (n=1,039 episodes; 305 in the clinic and 734 in the hospital)	PCR/DNA microarray hybridization assay Hospitalization was defined as admission for more than 24 hours to a short-stay unit, paediatric ward or PICU	RSV was the most frequently identified virus in infants and young children in hospital (n=467/734, 63.6%) with age younger than six months and prematurity associated with severe RSV cases among hospitalized children
Gilca *et al.,* 2020 ((11)) Canada (QC; Nunavik)	Retrospective cohort study	2012/11/01–2019/06/30 Children were followed up to one year of age or until 2019/06/30	Nunavik infants less than one year of age hospitalized for a respiratory illness (ICD-10 codes J00-J22 at any point, n=354)	RSVH was defined as hospitalization lasting 24 hours or longer with at least one positive RSV specimen collected during hospitalization or within four days prior to admission	113 (25%) of 458 episodes had RSV; annual average was 2.5 RSV-positive hospitalizations in high-risk infants and 16 RSV-positive hospitalizations in healthy full-term infants The overall RSVH rate per 1,000 live births in children younger than one year of age (adjusted for missed cases): • High-risk infants: 147.6 • Healthy full-term infants: 64.8 • Overall: 72.6
Piesky *et al.,* 2016 ((12)) Canada (ON)	Retrospective chart review	2010/01/01–2011/12/31	Children younger than three years of age residing within the Ottawa region potentially hospitalized for RSV (true positive cohort: n=1,119, and annual incidence estimates: n=19,815)	RSV hospitalization was defined as a positive test for RSV within 72 hours of admission and if the signs and symptoms responsible for hospital admission were consistent with RSV pathophysiology	Hospital admissions in children attributable to RSV: • Younger than one year of age: 8.8% • 1–2 years of age: 4.5% • 2–3 years of age: 2.7% Incidence of RSV hospitalization per 1,000 children from 2005 to 2012: • Younger than one year of age: 10.2 • 1–3 years of age: 4.8
Buchan *et al.,* 2023 ((13)) Canada (ON)	Population-based birth cohort study	First hospitalization in children born between 2009/05–2019/06	Children born May 2009 to June 2015 (n=826,140)	RSV hospitalizations were identified using a validated algorithm based on ICD-10 codes and/or laboratory-confirmed outcomes	12,573 (1.4%) incident cases of RSV hospitalization Rate of RSV-hospitalization per 1,000 patient-year (95% CI): • Range: from 29.55 (28.29–30.87) in children one month of age to 0.52 (0.47–0.57) in those 36–59 months of age • Overall: 4.23 (4.16–4.30) RSV hospitalization rates varied inversely with gestational age
McLaughlin *et al.,* 2022 ((14)) US	Systematic review and meta-analysis	Studies identified were published 2000–2020, and reported and collected 1989–2016	Children younger than five years of age (n=25 studies gave 31 estimates)	RSV hospitalization: • 13% (n=4/31) etiologic confirmation of RSV • 10% (n=3/31) clinician-directed standard-of-care medical and laboratory records • 65% (n=20/31) administrative claims data using RSV-specific ICD-9 codes • 13% (n=4/31) combined ICD-9 claims and etiologic surveillance data	Pooled rate of RSV-associated hospitalization per 1,000 (95% CI), n=31: • Younger than six months of age: 26.2 (24.2–28.2) • Younger than one year of age: 19.4 (17.9–20.9) • Younger than five years of age: 5.2 (4.8–5.6)
Stein *et al.,* 2017 ((15)) 32 countries (26 countries reported data on RSV-associated severe ARI hospitalization)	Systematic review and meta-analysis	Studies published 2000–2015	Children younger than five years of age not receiving RSV immunoprophylaxis with palivizumab (n=55 studies, of those 34 reported on hospitalization for severe RSV-ARI)	Case of severe ARI included hospitalized ARI or hospitalized lower or acute lower respiratory infection, pneumonia, and bronchitis	RSV-associated ARI hospitalization per 1,000 children-year (95% CI), (number of studies): • Younger than six months: 20.01 (9.65–41.31), n=6 • Younger than 12 months: 19.19 (15.04–24.48), n=18 • Younger than five years: 4.37 (2.98–6.42), n=15
Suh *et al.,* 2022 ((16)) US	Systematic review	Studies published 2000/01/01–2021/06/11 (data 1979–2020)	Studies of US infants younger than one year of age with clinical sequelae of RSV, and bronchiolitis (n=141 studies)	Lab-confirmed or ICD diagnostic codes for RSV hospitalization or bronchiolitis hospitalization	Five studies provided nationally representative data on annual average RSVH rates per year ranging from 11.6 (95% CI: 6.9–16.3) per 1,000 per year among infants 6–11 months of age to 50.1 (95% CI: 35.6–64.6) per 1,000 per year among infants 0–2 months of age
Wingert *et al.,* 2021 ((4)) OECD countries	Rapid review	Studies published 2014/01/01–2018/09/06	Children 24 months of age and younger, with or without a risk factor of interest, or immunocompromised children 18 years of age and younger without palivizumab prophylaxis with lab-confirmed RSV infection (n=29 cohort studies)	Lab-confirmed RSV-hospitalization, ICU admission, oxygen support, mechanical ventilation, extracorporeal membrane oxygenation, case fatality and complications from RSV infections (e.g., secondary infection)	RR (95% CI) RSV-hospitalization (number of studies): • 29–32 wGA vs. 33–36 wGA: 1.20 (0.92–1.56), n=1 • 33–36 wGA vs. ≥37 wGA: 2.05 (1.89–2.22), n=1 • Fewer than 33 wGA vs. 39–41 wGA: 3.88 (1.13–13.30), n=1
Li *et al.,* 2022 ((8)) World Bank income regions (data reported for high income)	Systematic review of studies published 2017/01/01–2020/12/31	2019 or before (i.e., before the onset of the COVID-19 pandemic)	Children 0–60 months of age	RSV-associated acute lower respiratory infection was defined as acute lower respiratory infection with lab-confirmed RSV infection RSV-attributable acute lower respiratory infection was defined as acute lower respiratory infection that could be causally attributable to lab-confirmed RSV-infection	Hospital admission rate per 1,000 children per year due to RSV-associated acute lower respiratory infection in high income countries (number of studies): • 0–3 months (n=19): 34.7 (21.5–56.2) • 3–6 months (n=21): 20.7 (13.5–31.6) • 0–6 months (n=27): 28.4 (20.2–40.0) • 6–12 months (n=27): 11.2 (7.5–16.7) • 0–12 months (n=41): 22.0 (17.1–28.4)
Bont *et al.,* 2016 ((17)) Western Countries (Canada, the US, and Europe)	Systematic review	Studies published 1995/01/01–2015/12/31	Children 18 years or younger	Hospitalization for RSV-related ARI or RSV-related bronchiolitis	RSV was associated with 19%–81% of all viral ARIs causing hospitalization Annual hospitalization rates per 1,000 children per year for RSV-associated ARIs: • 0–12 months: ranging from 3.2–42.7 • 1–4 years: ranging from 0.6–1.78 More than 70% of children hospitalized with RSV-associated ARIs had no underlying medical conditions Compared to influenza, RSV causes up to 16 times more hospitalizations and ED visits in children younger than five years
**RSV respiratory tract infection with intensive care unit admissions**					
Papenburg *et al.,* 2012 ((10)) Canada (QC)	Prospective cohort study	Four consecutive winter seasons (2006/07–2009/10)	Children aged 0–35 months presenting as outpatients to paediatric clinics or hospitalized for RTI (n=1,039 episodes; 305 in the clinic and 734 in the hospital)	PCR/DNA microarray hybridization assay Hospitalization was defined as admission for more than 24 hours to a PICU	63.6% (n=467) were RSV-positive hospitalization 5.2% (n=24/460) of hospital admissions for RSV had ICU admission (similar for hMPV)
Piesky *et al.,* 2016 ((12)) Canada (ON)	Retrospective chart review	2010/01/01–2011/12/31	Children younger than three years of age residing within the Ottawa region potentially hospitalized for RSV (true positive cohort: n=1,119)	RSV hospitalization was defined as a positive test for RSV within 72 hours of admission and if the signs and symptoms responsible for hospital admission were consistent with RSV pathophysiology	Of hospitalized cohort, 5.6% (95% CI: 5.2–5.9) were admitted to PICU and 3.1% (95% CI: 2.9–3.3) were intubated
Buchan *et al.,* 2019 ((18)) Canada (ON)	Retrospective multicentre cohort study	2009/05/01–2014/05/31	Hospitalized children aged 0–59 months tested for respiratory viruses including RSV (n=6,364)	Monoplex or multiplex PCR, viral culture or direct immunofluorescence	ICU admission: • 5% (n=192/3,569) with no comorbidities • 10% (n=275/2,795) if one or more comorbidity
Buchan *et al.,* 2023 ((13)) Canada (ON)	Population-based birth cohort study	First hospitalization in children born 2009/05–2019/06	Children born between May 2009 and June 2015 (n=826,140)	RSV hospitalizations were identified using a validated algorithm based on ICD-10 codes, and/or laboratory-confirmed outcomes	8.1% required intensive care during their hospitalizations (from 22% in those fewer than 28 weeks to 7% in those 37 weeks or more gestational age)
Amini *et al.,* 2019 ((19)) Canada (QC)	Prospective surveillance study	Peak weeks of five influenza seasons (2012/2013, 2014/2015–2017/2018)	Children younger than 24 months hospitalized with respiratory symptoms (n=546)	Multiplex PCR Hospitalization for 24 hours or longer for fever/feverishness or cough or sore throat	ICU admissions rates (*p*=0.07): • RSV: 3.6% • Influenza: 0%
Wildenbeest *et al.,* 2023 ((7)) Europe (five sites in Spain, Finland, England, Scotland and the Netherlands)	Multicentre, prospective birth cohort study	2017/07/01–2020/04/01	Healthy term infants, defined as children born 37 weeks or more of gestation with no evidence of significant disorders^a^, were included in the active surveillance cohort (n=993)	Parental questionnaire and hospital chart reviews, active RSV surveillance in nested cohort	Eight PICU admissions, corresponding to 5.5% of 145 RSV-associated hospitalizations and 0.09% of the total cohort Six of eight infants admitted to the ICU were younger than three months of age (median one month)
Suh *et al.,* 2022 ((16)) US	Systematic review	Studies published 2000/01/01–2021/06/11 (data 1979–2020)	Studies of US infants younger than one year of age with RSV, clinical sequelae of RSV and bronchiolitis (n=141 studies)	RSV and bronchiolitis defined as lab-confirmed and/or ICD codes	No studies reported nationally representative data. Twenty-two studies reported proportions of ICU admissions among RSV hospitalized infants (range: 6.3%–71.4%) Higher ICU admissions were observed in younger vs. older infants (up to 64.3% in those younger than six months vs. 54.5% in those six months and older; 2013–2016), preterm vs. full-term infants (52.2% vs. 33.3%; 1992–2017) From 2003 to 2007, 21.8% of infants with CHD and 13.3% of infants with CLD hospitalized for RSV had ICU admissions
**RSV respiratory tract infection with death**					
Schanzer *et al.,* 2018 ((20)) Canada (except QC)	Retrospective population-based study	September 2003 to August 2014 (nine influenza seasons, excluding the 2008/2009 and 2009/2010 seasons)	All patients admitted to an acute care hospital for a respiratory condition	Hospitalization with an ICD-10 code for RSV (J12.1, J20.5, J21.0, B97.4)	RSV-attributed inpatient death rate: 0.6 (95% CI: −0.1–1.3) per 100,000 population (not limited to paediatric)
Buchan *et al.,* 2023 ((13)) Canada (ON)	Population-based birth cohort study	First hospitalization in children born 2009/05–2019/06	Children born May 2009 to June 2015 (n=826,140)	RSV hospitalizations were identified using a validated algorithm based on ICD-10 codes and/or laboratory-confirmed outcomes	12,573 (1.4%) incident cases of RSV hospitalization A small proportion of those (0.2%) died within 30 days of discharge
Reichert *et al.,* 2022 ((21)) US	Population-based birth cohort study	1999–2018	All infants born to residents of the US and those who died at younger than one year of age with RSV, bronchiolitis or influenza as the cause of death (n=80,764,705 live births, 510,502 total infant deaths from all causes)	RSV was defined by at least one ICD-10 cause of death codes: B97.4 (RSV), J12.1 (RSV, influenza), J20.5 (acute bronchitis due to RSV) and J21.0 (acute bronchiolitis due to RSV)	The overall infant mortality rates from 1999 to 2018: • RSV: 6.9 (95% CI: 6.4–7.5) per 1,000,000 live births (n=561) • Bronchiolitis: 19.8 (95% CI: 18.9–20.8) per 1,000,000 live births (n=1,603) • Influenza: 6.2 (95% CI: 5.7–6.8) per 1,000,000 live births (n=504) Infant RSV mortality rates by birth year from 2008 to 2018 ranged from 8.1 (95% CI: 5.5–11.4) to 3.4 (95% CI: 1.9–5.7) per 1,000,000 live births Infant RSV mortality rates among younger than 29 wGA infants was 103.5 (95% CI: 81.8–129.1) RSV mortality burden was greatest in full-term (53.7%) infants
Li *et al.,* 2022 ((8)) World Bank income regions (data reported for high income)	Systematic review of studies published 2017/01/01–2020/12/31	2019 or before (i.e., before the onset of the COVID-19 pandemic)	Children 0–60 months of age	RSV-associated acute lower respiratory infection was defined as acute lower respiratory infection with lab-confirmed RSV infection RSV-attributable acute lower respiratory infection was defined as acute lower respiratory infection that could be causally attributable to lab-confirmed RSV-infection	Case fatality rate of in-hospital deaths in high-income countries for children 0–12 months with RSV-associated acute lower respiratory infection: 0.1% (95% CI: 0.1–0.3) (n=29 studies)

Abbreviations: ARI, acute respiratory infection; CHD, congenital heart disease; CI, confidence interval; CLD, chronic lung disease; COVID-19, coronavirus disease 2019; DNA, deoxyribonucleic acid; ED, emergency department; hMPV, human metapneumovirus; ICD, International Classification of Diseases; ICU, intensive care unit; NVSN, New Vaccine Surveillance Network; OECD, Organisation for Economic Co-operation and Development; ON, Ontario; PCR, polymerase chain reaction; PICU, paediatric intensive care unit; POCT, point-of-care testing; QC, Québec; RR, risk ratio; RSV, respiratory syncytial virus; RSVH, respiratory syncytial virus hospitalization; RT-PCR, reverse transcriptase polymerase chain reaction; RT-qPCR, quantitative reverse transcription polymerase chain reaction; RTI, respiratory tract infection; UR, uncertainty range; US, United States; wGA, weeks gestational age

^a^ Including cardiovascular, respiratory, renal, gastrointestinal, haematological, neurological, endocrine, immunological, musculoskeletal, oncological, or congenital disorders

**Medically attended RSV respiratory tract infection:** Three prospective observational studies demonstrated a high incidence of medically attended RSV infections. A US-based surveillance system from 2002 to 2004 found that RSV accounted for 18% of ED visits and 15% of office visits for ARI from November through April with higher rates in infants ((5)). More than 70% of the outpatients were previously healthy. Another US study from 2003 to 2005 reported 21.5 RSV-related ED visits per 1,000, higher than influenza (n=10.2 per 1,000), particularly in children younger than 24 months (n=64.4 visits per 1,000) ((6)). A European birth cohort in healthy term infants from 2017 to 2020 found a 26.2% (95% confidence interval [CI]: 24.0–28.6) RSV infection incidence and 14.1% (95% CI: 12.3–16.0) medically attended RSV incidence during the first year of life ((7)). Global data for children younger than five years aligned with these findings, reporting 38.5 (95% CI: 21.6–68.8) RSV-associated ARI per 1,000 children younger than one year of age in high-income countries ((8)).

Over five respiratory seasons, from 2018 to 2023, in Yukon, there were a total of 73 RSV infections in children 24 months and younger, which was higher than the number of influenza infections (n=20). Among infants younger than 12 months of age, the highest number of RSV infections occurred in those younger than three months of age. In summary, medically attended RSV infections are significant during infancy and early childhood, with approximately 10%–20% of infants seeking care for RSV in a season, surpassing medically attended influenza.

**Hospitalization associated with RSV respiratory tract infection:** Several Canadian studies highlight a substantial incidence of RSV-related hospitalizations in infants and young children. Schanzer *et al.*’s pan-Canadian study showed RSV as a major cause of hospitalization (n=130 per 100,000), with the highest rates in infants younger than six months ((9)). Papenburg *et al.*’s Québec-based study found RSV was the most common virus (63.6%) in children hospitalized for ARI, with higher severity linked to age under six months and prematurity ((10)). In Nunavik, RSV hospitalization rates were higher in high-risk infants (147.6 per 1,000 live births) compared to healthy term infants (n=64.8 per 1,000) ((11)). An Ontario study by Pisesky *et al.* reported RSV hospitalization rates of 10.2 per 1,000 children younger than one year and 4.8 per 1,000 in children one to three years of age ((12)). Buchan *et al.*’s Ontario cohort study revealed varying RSV hospitalization rates across age groups, with the highest in one-month-olds (n=29.55 per 1,000) and declining with age, with rates highest among children born at younger gestational ages ((13)). Over five respiratory seasons from 2018 to 2023 in Yukon, there were 27 severe RSV cases (non-ICU hospitalizations, ICU admissions and deaths), of which 18 were non-ICU hospitalizations. During that same time period, in children 24 months and younger, the number of severe RSV (n=27) cases was higher than the number of severe influenza cases (n=7).

Authors of systematic reviews and meta-analyses have also examined RSV hospitalization rates in infants and young children. McLaughin *et al.* reported US rates of 26.2 (95% CI: 24.2–28.2) and 19.4 (95% CI: 17.9–20.9) per 1,000 infants younger than six months and younger than 12 months, respectively ((14)). Stein *et al.* found rates of 20.01 (95% CI: 9.65–41.31) and 19.19 (95% CI: 15.04–24.48) per 1,000 children-years in the same groups ((15)). United States national studies reported annual RSV hospitalization rates ranging from 11.6 to 50.1 per 1,000 among infants ((16)). A rapid review showed varying incidence rates, from 1.2% in healthy term infants to 2.8%–5.1% in preterm infants ((4)). Global analysis found similar rates in high-income countries, with 28.4 (95% CI: 20.2–40.0) and 22.0 (95% CI: 17.1–28.4) per 1,000 in infants younger than six months and 12 months, respectively ((8)). A systematic review identified that RSV was associated with 19%–81% of all viral ARI causing hospitalization ((17)). While rates varied by a factor of 2–3 over seasons, they decreased significantly with increasing age. The majority (more than 70%) of children hospitalized had no underlying risk factors. Compared to influenza, RSV caused up to 16 times more hospitalizations in children younger than five years of age ((17)). In summary, RSV-related hospitalization rates vary by age and risk factors, with consistent trends of decreasing rates with increasing age. Despite vulnerability in high-risk groups, the majority of hospitalized children have no underlying medical conditions and RSV tends to lead to more hospitalizations compared to influenza.

**Intensive care unit admission associated with RSV respiratory tract infection:** Canadian studies indicate that approximately 5% of RSV-hospitalized children required ICU admission. Papenburg *et al.* found that 5.2% needed ICU care ((10)), while Pisesky *et al.* reported 5.6% ICU admission among RSV-hospitalized children younger than three years ((12)). Buchan *et al.* reported 5% ICU admission for healthy children younger than five years, increasing to 10% with comorbidities ((18)). In their 2023 study, ICU admission reached 8.1% among RSV-hospitalized children under five, with higher rates for premature births ((13)). In one Canadian study, ICU admission was more common with RSV compared to influenza ((19)).

International data align with this rate. A European birth cohort study in healthy term infants found 5.5% of RSV-associated hospitalizations led to ICU admissions ((7)). An SR of RSV disease in the US identified ICU admission proportions ranging from 6.3% to 71.4% and linked risk factors to younger age, prematurity, congenital heart disease and chronic lung disease ((16)). In summary, Canadian research suggests approximately 5% of RSV-hospitalized children required ICU admission, with higher rates among those with risk factors. In comparison to influenza, there is some evidence that RSV leads to more ICU admissions.

**Death associated with RSV respiratory tract infection:** Existing literature suggests a low risk of RSV-related mortality in both Canada and the US. An overall mortality rate of 0.6 per 100,000 population was reported by Schanzer *et al.*’s 2018 Canadian model of all patients in Canada admitted to hospital with a respiratory condition (from infancy to older than 65 years) ((20)). Buchan *et al.*’s 2023 Ontario cohort found a 0.2% mortality rate within 30 days of discharge from RSV hospitalization ((13)). In the US, an infant cohort followed from 1999 to 2018 showed a RSV mortality rate of 6.9 (95% CI: 6.4–7.5) per one million live births, with preterm infants at the highest risk ((21)); however, the majority of deaths occurred in full-term infants (53.7%), primarily those between one and four months of age (63.8%). Globally, a systematic analysis reported a 0.1% (95% CI: 0.1–0.3) case fatality rate for in-hospital RSV deaths in children 0–12 months of age ((8)).

### Pregnant women and people

**Figure 2:**
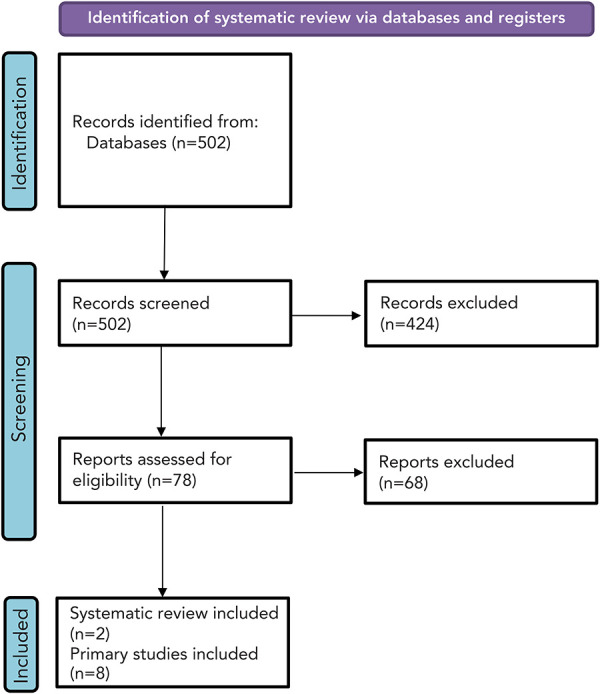
Study selection PRISMA flow diagram in pregnant women and people

**Study selection:** After removing duplications, 474 primary evidence studies and 28 systematic reviews underwent screening (**Figure 2**). In total, two systematic reviews and eight studies were included in the narrative synthesis of RSV burden in pregnant women and people (**Table 3**). No data on RSV-related mortality was identified in pregnant women and people.

**Table 3:** Summary of included studies on the burden of disease of respiratory syncytial virus in pregnant people

Author, year (reference), country	Study design	Study period	Population	Outcome definition	Results
**Medically attended RSV respiratory tract infection**					
Hause *et al.,* 2019 ((22)) US	Cross-sectional study	2015/11/03–2016/05/10	Pregnant women and people in their 2^nd^ or 3^rd^ trimester enrolled prospectively during their regular prenatal visits (n=155)	Lab-confirmed acute respiratory illness	Seven of 65 (11%) pregnant women and people with ARI at their initial enrollment and eight of 77 (10%) pregnant women and people with ARI during the study period (initial or re-enrollment) had PCR-confirmed RSV infection Four (50%) PCR-confirmed RSV ARI cases reported symptoms of a LRTI, one was hospitalized RSV had an attack rate of 10%–13% among ambulatory pregnant women and people receiving routine prenatal care during the respiratory virus season
Hause *et al.,* 2018 ((23)) US	Cross-sectional study	2015/10/01–2016/05/10	Pregnant women and people in their 2^nd^ or 3^rd^ trimester enrolled prospectively during their regular prenatal visits (n=155)	RSV infection was determined by PCR or serology	Of the 81 ARI cases, 52 (64%) respiratory pathogens were detected: The most frequently detected viruses were rhinovirus (n=22; 27%), coronavirus (n=14; 17%) and RSV (n=8; 10%) 12 patients had fever; 17 had symptoms of LRTI Of the seven cases with fever in the ALRTI group, three were RSV-positive (one had HRV coinfection) Of those patients with LRTI, two reported decreased fetal heart rate and one RSV-positive case was hospitalized for respiratory illness
**RSV respiratory tract infection with hospitalization**					
Regan *et al.,* 2018 ((24)) Australia, Canada (ON), Israel and the US	Retrospective database study	2010–2016	Pregnant women and people aged 18–50 years who were admitted to hospital with an ARFI (n=1,604,206 pregnant women and people)	A RSV-positive ARFI hospitalization was defined as a positive RT-PCR test result within three days of hospital admission	13,694 hospitalized acute respiratory tract/febrile illness; 846 tested for RSV and influenza 2.5% (n=21) tested positive for RSV 51% (n=430) tested positive for influenza Fewer than 1% tested positive for both influenza and RSV
Nowalk *et al.,* 2022 ((25)) US	Population-based retrospective aggregate cohort study	2015/09/01–2018/08/31	Adults ages 18–64 years, 65 years and older and including pregnant women and people (n=13,174 pregnant women and people)	Aggregate data used to determine population-based RSV hospital burden	RSV burden of hospitalization ranged from 0 to 808 per 100,000 pregnant women and people: • 2015–2016: no hospitalized cases of RSV among pregnant women and people • 2016–2017: 431 per 100,000 • 2017–2018: 808 per 100,000 Average burden from 2015 to 2018 of 620/100,000 in pregnant women and people which was higher than the burden for non-pregnant adults 18 years and older (n=320/100,000)
Hause *et al.*, 2021 ((26)) US	Retrospective case series	2010/08/01–2017/04/30	Pregnant women and people aged 14–49 years who tested positive for RSV and were hospitalized for RSV infection during pregnancy (n=10)	Variable	275,349 pregnant women and people; 1,057 tested for RSV; 25 (2%) tested positive; 10 hospitalized during pregnancy and tested positive within two weeks prior to or during hospitalization Diagnoses: pneumonia/atelectasis (n=5), upper respiratory tract infection (n=2), asthma exacerbation (n=2), respiratory failure (n=2), sepsis (n=2) Six had obstetrical complications (one exacerbation of pre-existing short cervix with preterm labour, three preterm contractions (two of which had co-infections), one induction for preeclampsia); one preterm birth; one ICU admission/mechanical ventilation
**RSV respiratory tract infection with intensive care unit admissions**					
Hause *et al.*, 2021 ((26)) US	Retrospective case series	2010/08/01–2017/04/30	Pregnant women and people whose pregnancy ended in live birth (n=10)	Hospitalization during pregnancy and positive RSV test by culture or PCR	275,349 pregnant women and people; 1,057 tested for RSV; 25 (2%) tested positive; 10 hospitalized during pregnancy and tested positive within two weeks prior to or during hospitalization One of 10 (10%) required ICU admission and mechanical ventilation
Wheeler *et al.*, 2015 ((27)) US	Case series	Winter 2014	Antepartum RSV infection treated at single tertiary care facility (n=3)	N/A	Two of three cases required ICU admission and mechanical ventilation; all three cases complicated by pre-existing lung conditions (asthma, comorbid influenza, group A streptococcus infection)
Deshmukh *et al.*, 2014 ((28)) UK	Case report	Not stated	40-year-old pregnant person (n=1)	N/A	Pregnant person admitted to hospital in UK requiring ICU admission, mechanical ventilation, and emergency C-section at 33 weeks for maternal reasons (RSV pneumonitis and sepsis)
**Preterm labour/birth with RSV infection**					
Regan *et al.*, 2018 ((24)) Australia, Canada (ON), Israel and the US	Retrospective database study	2010–2016	Pregnant women and people 18–50 years of age who were admitted to hospital with an ARFI (n=1,604,206 pregnant women and people)	A RSV-positive ARFI hospitalization was defined as a positive RT-PCR test result within three days of hospital admission	13,694 hospitalized acute respiratory tract/febrile illness; 846 tested for RSV; 2.5% (n=21) tested positive by RT-PCR No difference in preterm, small-for-gestational age and low birth weight births between RSV-positive and RSV-negative participants Association observed between RSV positivity and subsequent preterm birth (*p*=0.034): • RSV-positive participants, 29% • RSV-negative participants, 15%
Chu *et al.*, 2016 ((29)) Nepal	Prospective randomized trial	April 2011–May 2014	Pregnant women and people in the 2^nd^ trimester of pregnancy and followed until six months postpartum (n=3,693; 14 RSV illness episodes over 3,554 person-year surveillance)	RSV-positive tests were determined by rt-PCR	Seven (50%) pregnant participants sought care for RSV illness; none died Of the seven (50%) illness episodes during pregnancy, all had live births with two (29%) preterm births and a median birth weight of 3,060 grams. This compares to 469 (13%) preterm births and a median birth weight of 2,790 grams in persons without RSV during pregnancy
Hause *et al.*, 2021 ((26)) US	Retrospective case series	2010/08/01–2017/04/30	Pregnant women and people whose pregnancy ended in live birth (n=10)	Hospitalization during pregnancy and positive RSV test by culture or PCR	275,349 pregnant women and people; 1,057 tested for RSV; 25 (2%) tested positive; 10 hospitalized during pregnancy and tested positive within two weeks prior to or during hospitalization One of 10 (10%) participants had pneumonia and preeclampsia and was induced between 36 and 37 weeks

Abbreviations: ALRTI, acute lower respiratory tract infection; ARFI, acute respiratory infection or febrile illness; ARI, acute respiratory infection; HRV, human rhinovirus; ICU, intensive care unit; LRTI, lower respiratory tract infection; N/A, not applicable; ON, Ontario; PCR, polymerase chain reaction; RSV, respiratory syncytial virus; rt-PCR, real-time polymerase chain reaction; RT-PCR, reverse transcription polymerase chain reaction; UK, United Kingdom; US, United States

**Medically attended RSV respiratory tract infection:** Two US cross-sectional studies by Hause *et al.* investigated RSV infection rates in pregnant women and people in their second or third trimester during the 2015–2016 RSV season. In one study, with combined PCR and serological data, the RSV attack rate among ambulatory pregnant women and people receiving routine prenatal care was estimated at 10%–13% ((22)). In the second study, approximately 10% of acute lower respiratory tract illness cases in pregnant women and people were confirmed as RSV ((23)).

**Hospitalization associated with RSV respiratory tract infection:** The literature on RSV-associated hospitalizations presents a broad range of rates. A retrospective study within the Pregnancy Influenza Vaccine Effectiveness Network (PREVENT) 2010–2016 found a 2.5% RSV-positive rate, contrasting with a 51% influenza-positive rate ((24)). A US population-based study from 2015 to 2018 revealed higher hospitalization rates among pregnant women and people compared to non-pregnant adults (average rate of 620 vs. 320 per 100,000) ((25)). Additionally, one retrospective case series documented adverse pregnancy outcomes in ten pregnant individuals hospitalized with RSV, including pneumonia, respiratory failure and sepsis, with six experiencing obstetrical complications during hospitalization, including preterm contractions, coinfections and preeclampsia ((26)). In summary, the literature suggests a wide range of possible RSV hospitalization rates among pregnant women and people, with one study indicating a higher burden compared to non-pregnant adults.

**Intensive care unit admission associated with RSV respiratory tract infection:** Evidence on RSV-related ICU admissions is limited. In a retrospective case series focusing on adverse pregnancy outcomes, one of 10 pregnant women and people required ICU admission and mechanical ventilation ((26)). Another case series of three pregnant women and people with RSV found that two required ICU admission and mechanical ventilation, while the third was monitored as an outpatient ((27)). A case report describes a pregnant person admitted with RSV pneumonitis and sepsis, requiring ICU admission, mechanical ventilation and emergency C-section ((28)). However, data regarding the risk of ICU admission among pregnant women and people remain scarce.

**Outcome for both infants and pregnant women and people—preterm labour/birth:** Three studies reported data on the risk of preterm labour/birth associated with RSV infection. In the Pregnancy Influenza Vaccine Effectiveness Network study, no difference was observed in preterm, small for gestational age, and low birth weight births between RSV-positive and RSV-negative pregnant women and people ((24)). However, among ARI admissions without delivery during the hospital admission, RSV positivity was associated with subsequent preterm birth (29% vs. 15%). A study from Nepal showed a higher rate of preterm birth with RSV illness episodes during pregnancy (29% vs. 13%) ((29)). In a case series of ten pregnant women and people hospitalized with RSV, one had preterm birth (10%) ((26)). In summary, available evidence is insufficient to assess the risk of preterm labour/birth due to RSV infection during pregnancy.

## Discussion

This rapid review offers insight into RSV burden in predominantly high-income countries, with a focus on Canada, the US and Europe. More robust evidence was available for infants and young children, with Canadian studies contributing significantly, while evidence for pregnant women and people primarily stemmed from small observational studies outside Canada. In infants and young children, medically attended RSV was common, and RSV hospitalization rates varied but generally decreased with age. Most hospitalized children had no underlying medical conditions. Approximately 5% of RSV-hospitalized children in Canada required ICU admission, and the risk of death was low. Respiratory syncytial virus caused a higher burden of disease than influenza in this population. Novel and previously unpublished data from the Yukon support the conclusions of this literature review, noting a higher burden of RSV than influenza and the highest burden in younger age groups. For pregnant women and people, RSV severity appeared to be similar to non-pregnant women and people, with an attack rate of 10%–13% during the respiratory virus season. One study reported higher RSV hospitalization rates than those for non-pregnant women and people. Data on ICU admission, death and preterm birth related to RSV in pregnancy were limited, although two studies suggested an association with preterm birth.

This rapid review highlights limitations in characterizing RSV burden in Canada. Studies often focused on RSV-associated hospitalization and ICU admission, which are critical outcomes for assessing severe clinical consequences. However, it is also essential to grasp the significance of other outcomes in the Canadian context, in particular medically attended RSV and death related to RSV infection. Currently, Canada has limited enhanced national RSV surveillance data. Recent research initiatives have leveraged existing healthcare administrative databases to characterize RSV burden; however, those data are expected to underestimate RSV disease especially in the community and outpatient setting due to undertesting in routine clinical care, the lack of generalizability to the Canadian population and healthcare coding systems that do not capture all possible contributors to RSV-related complications ((30)).

## Limitations

This rapid review has limitations. It primarily focused on short-term outcomes and did not consider potential long-term effects such as asthma, which may be associated with early-life RSV infection ((31,32)). Detection of RSV infection was not limited to laboratory confirmation; some studies relied on clinical diagnostic codes, potentially inflating RSV incidence. Estimates were imprecise. Robust data on severe RSV outcomes in pregnant women and people were lacking; however, historically, pregnant women and people have not been known to specifically be at higher risk of RSV infection. Although the goal of forthcoming RSV immunization products is to reduce complications of RSV in infants, it is essential to also consider the potential benefits of a RSV vaccine for pregnant people, given their increased susceptibility to certain respiratory pathogens such as influenza resulting from pregnancy-related changes in anatomy and the immune and cardiovascular systems. This review did not specifically focus on RSV burden during the coronavirus disease 2019 (COVID-19) pandemic. The public health measures in place during the early phase of the pandemic led to a significant reduction of seasonal respiratory virus circulation ((33)). In recent seasons, there has been a substantial increase in RSV cases, with changes in age distribution and atypical seasonality patterns compared to prior to the COVID-19 pandemic, attributed to larger populations of RSV-naive children (34,35)). For example, a recent publication from 13 paediatric centres in Canada noted a significant burden of RSV hospitalizations, with a significant increase in hospitalizations in 2021–2022 compared to pre-pandemic ((36)). Despite these limitations, the data presented here provide a foundation for understanding the typical RSV burden in infants and young children.

## Conclusion

A high incidence of medically attended RSV is observed in infants and young children, with hospitalization rates decreasing with age. Approximately 5% of hospitalized infants and young children with RSV required ICU admission. The risk of death appears to have been low. Pregnant and non-pregnant women and people showed similar RSV severity, although data were limited for pregnant individuals. With the introduction of interventions, RSV’s disease burden is expected to change; robust surveillance systems at the provincial, territorial and national levels will be crucial for evaluating the public health impact of RSV immunization programs. This review contributes to the literature, aiding in characterizing RSV’s burden in Canada and guiding RSV immunization strategies for infant protection.

## Supplemental material

These documents can be accessed on the Supplemental material file.
